# Plaque-associated lipids in Alzheimer’s diseased brain tissue visualized by nonlinear microscopy

**DOI:** 10.1038/srep13489

**Published:** 2015-08-27

**Authors:** Juris Kiskis, Helen Fink, Lena Nyberg, Jacob Thyr, Jia-Yi Li, Annika Enejder

**Affiliations:** 1 Department of Biology and Biological Engineering, Chalmers University of Technology, Göteborg, Sweden; 2 K-Analys, Salagatan 16 A, Uppsala, 753 30, Sweden; 3 Department of Experimental Medical Science, Lund University, Lund, Sweden

## Abstract

By simultaneous coherent anti-Stokes Raman scattering (CARS) and 2-photon fluorescence microscopy of Thioflavin-S stained Alzheimer´s diseased human brain tissues, we show evidence of lipid deposits co-localizing with fibrillar β-amyloid (Aβ) plaques. Two lipid morphologies can be observed; lamellar structures and coalescing macro-aggregates of sub-micron sizes to ~25 μm. No significant lipid deposits were observed in non-fibrillar, diffuse plaques identified by Aβ immuno-staining. CARS microscopy of unlabeled samples confirms the lamellar and macro-aggregate lipid morphologies. The composition of the plaques was analyzed by CARS microspectroscopy and Raman microscopy; vibrational signatures of lipids with long acyl chains co-localize with the β-sheet vibrations. The lipid fluidity was evaluated from the CARS spectra, illustrating that the lipid composition/organization varies throughout the plaques. Altogether this indicates close amyloid-lipid interplay in fibrillar Aβ plaques, rendering them more dynamic compositions than previously believed and, hence, potential sources of toxic oligomers.

Alzheimer’s disease (AD) is estimated to be the fourth-leading cause of death in high income countries and the only lethal disease among the top ten that currently has no means for cure, prevention or even delay^1^. Hence, the prevalence of AD continues to increase with 7.7 million new cases diagnosed world-wide every year and is expected to escalate further with the aging population^2^. Early pathological hallmarks are the formation of soluble β-amyloid (Aβ) oligomers and synaptic dysfunction, believed to cause mild cognitive impairment long before the accumulation of amyloid plaques and neurodegeneration^3^,^4^,^5^,^6^,^7^,^8^. Aβ oligomers have been shown to bind to cellular membranes in human cortical neurons with high affinity, and more specifically to synaptic contacts^9^. Here they disrupt the membrane barrier function by *e.g.* altering the membrane structure, complete membrane rupture or forming pores, followed by Ca^2+^ influx and accumulation of reactive oxygen species (for a review, see Stefani^10^). Aβ oligomers have also been shown to specifically bind to post-synaptic receptors, in turn modulating down-stream signaling pathways causing alterations in the axonal transport of synaptic vesicles and mitochondria leading to synaptic impairment (for reviews, see Larson *et al.*^11^ and Overk *et al.*^12^). In the advanced stages of AD, Aβ oligomers accumulate to insoluble plaques, surrounded by a distinct halo of free Aβ oligomers^13^. Neuroinflammation and activation of the mitochondrial apoptotic pathway^14^,^15^ result in manifest neurodegeneration, the degree of which correlates well with the gradient distribution of free oligomers in the halo and the surrounding neuropil^13^. It is proposed that the Aβ oligomers in the halo are released by the fibrils in the plaque^13^, which is supported by *in vitro* studies showing that Aβ fibrils continuously dissolve and reform^16^, rather than being irreversibly trapped as inert fibrous species. Lipids interacting with amyloid fibrils have been shown to significantly promote the disintegration into toxic oligomers by acting as detergents^17^,^18^. Interestingly, there is a line of evidence that fibrillar plaques contain lipids^19^,^20^,^21^,^22^,^23^,^24^, hence, boosting the release of oligomers and rendering them a huge reservoir of toxic species. This lipid-promoted fibril fragmentation is further confirmed by molecular dynamics simulations^25^. Altogether, these studies underline that Aβ oligomers are the active players in AD, however, also that their dynamics and distribution is strongly modulated by their interaction with lipids. While analytical methods and fluorescence microscopy^26^,^27^,^28^ allow us to obtain important quantitative and spatial information on Aβ species alone, a more holistic understanding of the underlying chain of mechanisms could be gained by taking into account their close interplay with lipids. Such information is currently almost non-existing, partly due to the lack of reliable methods to detect as fragile entities as lipids; their organization and distribution are highly influenced by changes in their physicochemical environment in conjunction with *e.g.* extraction procedures, sample preparation and labeling. Lipidomic analyses, involving chemical extraction of lipids from larger tissue samples and plasma, reveal elevated ceramide, cholesterol and triglyceride levels in conjunction with amyloid deposition^29^, though without any information on the spatial distribution in the brain. Fluorescence microscopy suggests increased accumulation of cholesterol in senile plaques^30^,^31^, but these results have been shown to originate from false-positive staining^32^. Time-of-flight secondary ion mass spectrometry (ToF-SIMS) imaging reports a slight increase in the overall cholesterol signal in the cortex of human AD brain tissue compared to controls^33^. Minor depositions of cholesterol granules were observed immediately around the plaques in an AD mice model^24^, however, the signals were comparatively weak and the granular distribution could in a follow-up study not be confirmed^34^. Altogether, no significant lipid deposits are reported by ToF-SIMS, when studying a shallow (10 nm) two-dimensional plane of the surface (sensitive to influence of topographic variations) and to the low m/z region. No data in the high m/z region *e.g.* triglycerides seem to be available in the current literature to the best of our knowledge. Fourier transform infrared (FTIR) microscopy is capable of simultaneous imaging of lipids and amyloid proteins, revealing elevated lipid concentrations in regions identified as core plaques in transgenic mouse models and human AD brain tissues^19^,^22^,^23^. Due to the low number of pixels covering the plaque region, each pixel representing ~5 μm, it is difficult to conclude any detailed information on the lipid distribution and to what extent lipids co-localize with amyloid fibers. Altogether, there are convincing data that there are accumulations of lipid in the vicinity of fibrillar Aβ plaques, but no detailed information on the morphology of the lipid deposits, on their fluidity (*i.e.* stability) or degree of co-localization with the Aβ fibrils – all important information in order to better understand the underlying accumulation process(es) and their ability to release toxic oligomers.

In this study we use the strengths of multimodal non-linear optical (NLO) microscopy, combining coherent anti-Stokes Raman scattering (CARS) and multiphoton excited fluorescence (MPEF), for simultaneous investigations of spatial distributions of lipids and fluorescently stained Aβ fibrils in *human* AD brain tissue sections in order to complement with more detailed information. CARS microscopy has been previously shown to be a valuable label-free tool to visualize lipids in other tissues and cells by exciting and probing CH stretching vibrations of fatty acids via a nonlinear optical wave-mixing process^35^,^36^,^37^,^38^,^39^. In the current study, images of the distributions of lipids in AD brain tissue are complemented by MPEF images of the Aβ fibrils labeled with Thioflavin-S^40^. Three-dimensional renderings of the lipid/Aβ fibril distributions are presented at high spatial resolution (~0.3 μm in x/y and ~1 μm in z), allowing us to resolve regions with distinct different contents of lipids and Aβ fibrils within and around core plaques and assess to what extent they co-localize. We complement the data by *label-free* imaging using CH-CARS only (no Thioflavin-S staining), as well as Raman microscopy in the CH vibrational region (lipids) and the amide vibrational region (Aβ species), in order to avoid possible labelling effects on the distribution/composition of the fragile lipids/amyloid co-arrangements. In contrast to previous FTIR and ToF-SIMS imaging studies, we present *simultaneously collected* images of *lipids* and *Aβ species* in *soft, wet tissue samples*, allowing us to directly relate the distributions without *e.g.* freeze-drying. Furthermore, by pixel-wise forming a ratio of the CARS signal at the symmetric and asymmetric CH vibrations, maps of the lipid fluidity (degree of unsaturation) throughout the plaque formations and surrounding regions are formed. This is of high interest, as the lipid environment controls the degree of fibril fragmentation; polyunsaturated lipids with a capability to form micelles are particularly efficient in preventing the aggregation of fibrils, leaving the Aβ species to a high degree as toxic protofibrils^41^. This kind of visual information can be used to assess to what extent the plaques are capable of releasing toxic oligomers, hence, their neurotoxicity.

## Results

**Figure 1:**
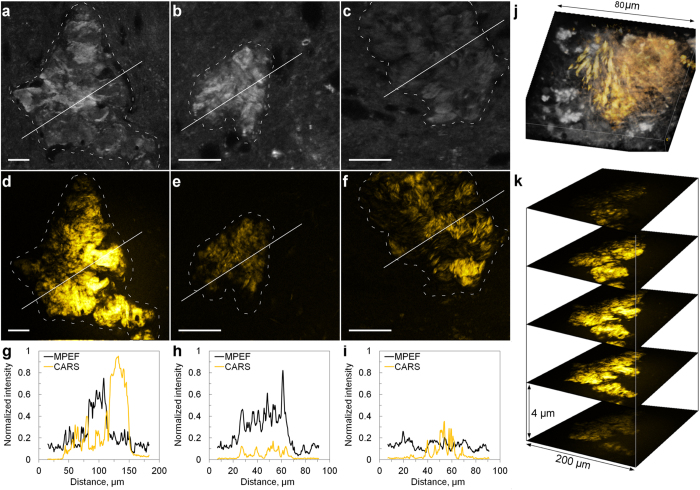
Visualization of Aβ and lipid distributions in core plaques by fluorescence/CARS microscopy. (**a–c**) Thioflavin-S fluorescence; (**d–f**) CARS images at 2840 cm^−1^; (**g–i**) profile plots along the lines indicated in (**a,d**), (**b,e**) and (**c,f**) respectively; (**j**) a 3D rendering of a CARS microscopy image (yellow) of lipids distributed in the AD plaque region, visualizing the different morphologies: crystalline needle-like structures (left) and more diffusely distributed (right); (**k**) z-stack representation of the CARS signal from the plaque in (**d**). Scale bars, 25 μm.

Tissue sections from the prefrontal cortex of AD diseased human brains were investigated. Fibrillar Aβ plaques were identified by MPEF from Thioflavin-S stained tissue sections (Fig. 1a–c). Simultaneously, the CARS signal was detected by tuning to the symmetric CH vibrational mode at 2840 cm^−1^, characteristic of long-chained triglycerides^42^. The CARS images revealed lipid accumulations primarily in the areas of fibrillar Aβ plaques (Fig. 1d–f). In general, core Aβ plaques contained higher Thioflavin-S and lipid concentrations on a macro scale (~100 μm) compared to the surrounding tissue (dashed outlines in Fig. 1a–f). However, within the plaque region, Thioflavin-S and lipid concentrations showed little correlation. Plaque areas with comparatively high fluorescence signals could be associated with both high and low lipid signals (Fig. 1g–i). Variations in the lipid distribution at different depths in the tissue could also be observed at the spatial resolution of 1 μm. A 3D rendering and a z-stack representation of the lipid distribution in a plaque is exemplified in Fig. 1j,k. Excitation beams could readily penetrate through the whole thickness of a tissue section (~40 μm).

In contrast to fibrillar Aβ plaques (Thioflavin-S positive), diffuse plaques (non-fibrillar; Thioflavin-S negative, here identified by Aβ immuno-staining) did not exhibit any significant lipid deposits (Supplementary Fig. S1 online). Further systematic studies are needed to determine whether fibrillar Aβ plaque content is prerequisite for lipid accumulation, or whether there are also classes of Aβ oligomers that co-localize with lipids.

**Figure 2:**
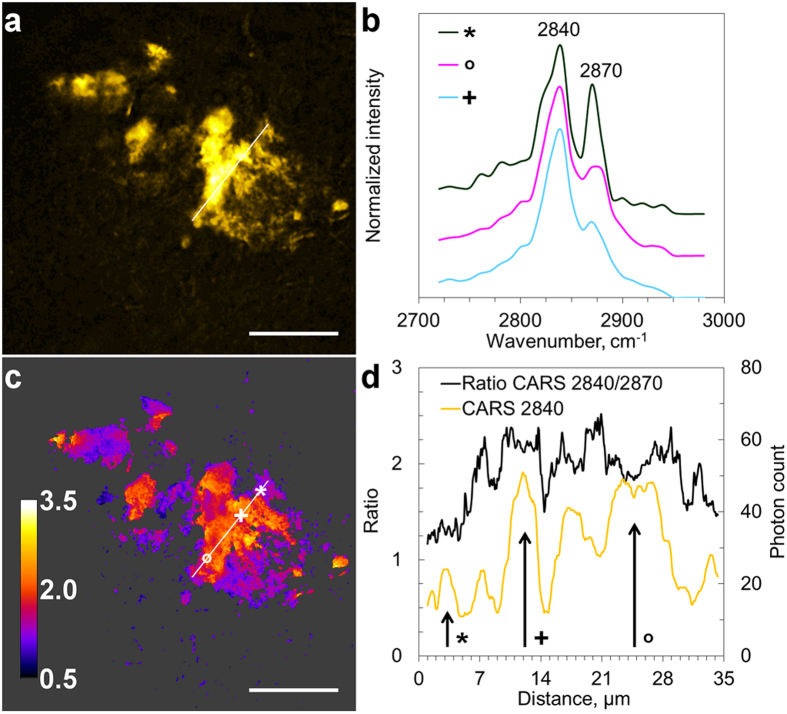
Visualization of the heterogeneity in lipid aggregates composition in AD brain tissue. (**a**) CARS (2840 cm^−1^) image of lipid aggregate; (**b**) CARS spectra collected at positions marked by symbols in **c**, curves are displayed with an offset for clarity; (**c**) CARS ratio image, intensities at 2840 cm^−1^ vs. 2870 cm^−1^, representing variations in lipid fluidity; (**d**) profile plots along the lines indicated in (**a,c)**. Scale bars, 25 μm.

In order to avoid possible interference from the labeling on the lipid distribution, we further studied unstained AD diseased human brain tissue sections. These sections were adjacent to samples with core plaques identified by Thioflavin-S staining. Detailed information on the lipid composition in the unstained samples was acquired by scanning CARS spectra in the CH-stretching vibration region (2720–2980 cm^−1^). Spectra of lipid aggregates in AD brain tissue, shown in Fig. 2a, were dominated by two peaks at 2840 cm^−1^ and 2870 cm^−1^ (Fig. 2b), corresponding to the symmetric and asymmetric CH_2_ vibrations, respectively. The spectral shape is similar to that obtained for lipids with long acyl-chains, however, spatial variations were observed (Fig. 2b), and minor contributions from *e.g.* cholesterol^43^ cannot be excluded.

Spectra collected at different spatial positions revealed notable differences in chemical composition, observed as different contributions from the two CH_2_ vibrations (Fig. 2b). In order to investigate the heterogeneity of the lipid composition within the lipid aggregates, ratios of CARS signals at 2840 cm^−1^ and 2870 cm^−1^ were calculated (Fig. 2c), providing an estimate of lipid fluidity^44^,^45^. Higher 2840 cm^−1^/2870 cm^−1^ ratios indicate lower packing of acyl chains, higher fluidity and/or more unsaturated lipids promoting Aβ fibril fragmentation^41^. Variations in the 2840 cm^−1^/2870 cm^−1^ ratio within the lipid aggregates suggest that they are complex and composed of areas with different lipid compositions with different propensity to release toxic Aβ oligomers.

**Figure 3:**
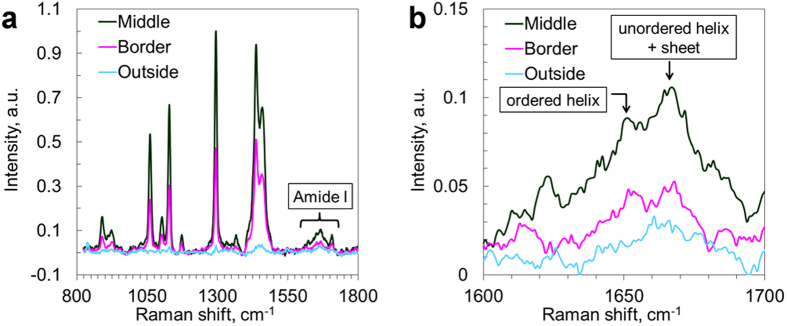
Raman spectra from lipid aggregates in human AD brain tissue. (**a**) Raman spectra in the finger print region highlighting the Amide I band; (**b**) Zoom-in on the Amide I band, from (**a**). Spectra were collected in the center and on the border of lipid aggregate, as well as from the tissue outside the lipid aggregate.

In order to investigate the contents of unlabeled samples, avoiding the potential impact of the labeling agent, we further complemented our data with Raman micro-spectroscopy (Fig. 3) in the fingerprint region, focusing in particular on the range 1620–1670 cm^−1^ - the amide I vibrations^46^. Spectra from the central part of the lipid aggregates (confirmed by strong CH vibrations) in AD brain tissue exhibited a distinct peak at 1667 cm^−1^ (β-sheet and unordered α-helix) and a small shoulder at 1655 cm^−1^ (ordered α-helix)^47^, in contrast to a featureless amide I band in the spectra from the surrounding tissue. In the plaque border region, the β-sheet peak could still be distinguished, but it was significantly weaker relative to the α-helix peak than in the core region. This indicates a higher amount of β-sheet formations in the lipid-rich areas compared to the surrounding brain tissues and confirms that the lipid aggregates indeed co-localize with Aβ species in AD brain tissues^48^. Furthermore, the overall Raman scattering intensities in the amide region collected in the center of the aggregate were significantly higher than at the border of and outside the aggregate, indicating that higher amounts of proteins could in general be found in the lipid deposit region. In order to identify which classes of lipids were present in the lipid aggregates, the fingerprint Raman micro-spectroscopy data were compared with spectra collected on different brain lipids as presented by Krafft *et al.*^49^. The shape of the spectra collected on the plaque-associated lipids (Fig. 3) was found to agree well with that of long-acyl chain fatty acids, hence, likely to be the main lipid component of the aggregates. The ester bond vibrations at 1729 and 1744 cm^−1^ are lacking as well as the phosphate group vibration at 860 cm^−1^, which suggests that triglycerides and phospholipids are minor components. Furthermore, the numerous sharp bands between 400 and 1200 cm^−1^, characteristic for cholesterol, could not be distinguished, which agrees with the observations made in the high-frequency CH vibrational region, *i.e.* minor contents of cholesterol.

**Figure 4:**
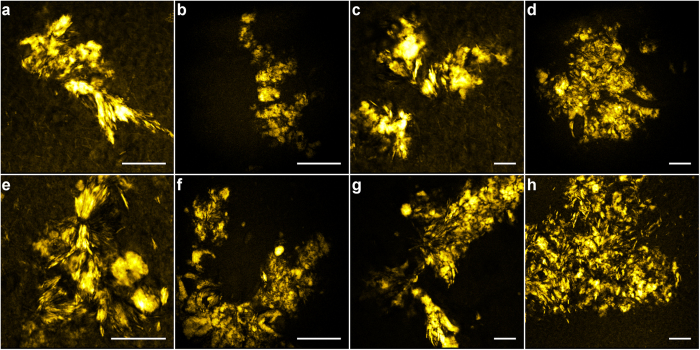
Differences in lipid aggregate morphology found in human AD brain tissues investigated by CARS microscopy (2840 cm^−1^) including two main categories; lamellar structures (*e.g.* in a, e and g) and coalescent structures of different sizes, possibly originating from lipid micelles initially decorating the Aβ fibrils or ApoE particles, alternatively from lipid microvesicles shedded by microglia (see discussion). (**a–d**) Patient №1; (**e–h**) patient №2. Scale bars, 25 μm. Note the different scales in the images.

Characteristic morphologies of lipid aggregates are observed and can be recognized in CARS images of tissue samples at different locations and from different patients; Fig. 4(a–d) patient №1, Fig. 4(e–h) patient №2. Some lipid aggregates seem to form lamellar structures, suggesting that lipids organize along and possibly separate Aβ species (Fig. 4a,e,g); some are composed of larger, coalescent structures (Fig. 4b,c,e), while others are formed from multiple, smaller domains (Fig. 4d,f–h). Sizes range from sub-micron to ~25 μm.

## Discussion

Biochemical analyses of lipid extracts from AD brain tissues and plasma^29^, as well as FTIR microscopy of brain tissue sections with fibrillar Aβ plaques^19^,^20^,^22^,^23^, report solid evidence for altered lipid composition and manifest lipid accumulations, respectively. However, more detailed information on the morphologies of the lipid deposits and their possible co-arrangements with Aβ species are needed to be able to find plausible underlying mechanisms. Information on the degree and character of Aβ/lipid co-arrangements in AD brain tissues are also important to sort out the conflicting reports on the toxicity of fibrillar plaques, taking into consideration the observations that lipid-decorated fibrils are readily destabilized and fragmented into toxic Aβ oligomers in contrast to pure, non-lipidated Aβ fibrils which are largely inert^18^. With the increased spatial resolution and three-dimensional imaging capability of NLO microscopy, our data reveal a morphologically and chemically more complex structure of Aβ fibrillar plaques than previously been reported. In the CARS microscopy images we note that lipids arrange as aggregates of primarily two different characters; (A) lamellar structures, as if templated by fibrillar Aβ species, and (B) coalescent macro-aggregates of various sizes and shapes (see examples in Fig. 1 and Fig. 4). These two arrangements could be found in labelled (Fig. 1) as well as in unlabeled samples (Fig. 2 and Fig. 4), confirming that they were not caused or affected by the labelling. Several different mechanisms underlying the formation of lipid lamella and coalescent regions are plausible. Electron microscopy has shown that Aβ fibrils actively extract lipids from vesicle membranes through a detergent-like mechanism^50^. When multiple protein and membrane segments interact under amyloid promoting conditions, it has been shown that they form multi-lamellar suprastructures^51^. The protein assemblies line up with lipids in-between and the positively charged residues connect to the negatively charged lipids. These mechanisms could explain the formation of the lipid lamella. Hellstrand *et al.* also report that α-synuclein form fairly aligned bundles of fibrils at no or low concentrations of lipids, however, a random network is rather established at higher lipid concentrations with excess lipids being trapped as deformed vesicles in between the fibrils^52^. The latter organization with lipid vesicles could potentially result in the coalescence to lipid macro-aggregates of different sizes, as we observe in the CARS microscopy images. Hence, the formation of lipid aggregates of two different characters might be due to local differences in lipid concentrations; indeed, the lipid droplet regions typically generate a stronger CARS signal than the lamellar structures. Several other mechanisms could explain the formation of coalescing lipid deposits. Microvesicles budding off from microglia have been shown to adhere to fibrillar plaques and both feed them with and extract toxic Aβ oligomers^53^. Also the lipid transporter protein Apolipoprotein E (ApoE) and its lipid cargo have been shown to co-localize with fibrillar Aβ plaques^31^. Both microglial lipid vesicles and ApoE show no affinity to Aβ oligomers^53^,^54^, which could explain why no lipid deposits are observed in diffuse plaques consisting of Aβ oligomers.

The proposed mechanisms suggest that the lipids in the aggregates observed in the CARS microscopy images originate from cell/organelle membranes, alternatively lipids carried by the ApoE complex (primarily cholesterol but also phospholipids^55^). Analytical data indeed report elevated levels of cholesterol in senile plaques^29^. FTIR spectroscopy instead suggests that the many CH and CO-related absorption peaks can be attributed to phospholipids, however, due to overlapping features no conclusive interpretations of the contents could be made more than the presence of lipids with long acyl chains^19^. Our Raman and CARS spectra confirm the FTIR data that the primary content is lipids with long acyl chains, which could mean *e.g.* fatty acids or ceramides. We hardly see any signs of the phosphate-group vibration at 860 cm^−1^, indicating no/small amounts of phospholipids. Furthermore, no signs of the ester-vibrations at 1729 and 1744 cm^−1^, characteristic of triglycerides, can be noted. However, this may be due to the low number of phosphate- and ester groups relative to the number of CH bonds per lipid molecule. Furthermore, we cannot exclude the presence of cholesterol, as its spectral features in the high-wavenumber CH region partly overlap with that of other lipids. For more specific characterization of the lipid content in the plaque-associated lipid aggregates, it would be interesting to complement with ToF-SIMS measurements in the high m/zregion.

The CARS spectra and CARS CH ratio image in Fig. 2 reveal that the composition of the lipids varies throughout a plaque. Data indicate that the region characterized by larger coalescent lipid aggregates consist of lipids with higher fluidity, whereas the lamellar regions pinched by the protein-lipid interaction sites consist of more ordered lipids. More thorough investigations are needed in order to interpret these variations. Furthermore, it would be interesting to compare the degree of lipid fluidity with the distribution of different Aβ species by immunostaining and fluorescence microscopy, in order to confirm previous observations that lipids with higher fluidity and a capability of forming micelles have a higher tendency to destabilize the Aβ fibrils to toxic Aβ oligomers^41^. With this knowledge the CARS CH ratio images could be used as indirect toxicity maps, requiring no immunostaining.

*In conclusion*, we have shown that a sub-population of fibrillar plaques co-localizes with lipid deposits, which consist of lipids with long acyl chains organized as either multi-lamellar structures or as collections of coalescent lipid aggregates of sizes from sub-micron to ~25 μm. No lipid deposits have been observed in diffuse plaques, lacking fibrillar Aβ. We show that the lipid composition in the lipid deposits, here in terms of the lipid fluidity, varies throughout the plaque region; important information as it determines to what extent toxic Aβ oligomers could be released from the fibrillar plaques. This study demonstrates the strength of NLO microscopy for simultaneous characterization of three-dimensional distributions of lipids and Aβ species under biologically relevant conditions in human AD brain tissues, illustrating that the chemical build-up of AD plaques is more complex than previously reported. It further allows us to study the intricate interplay between lipids and amyloids, necessary to deepen our understanding of the toxic mechanisms of Aβ plaques.

## Materials and Methods

### AD brain tissue samples

Human brain samples from two different individuals were obtained with informed consent from the families and with the ethical approval of institutional Ethics Committees from New York Brain Bank at Columbia University (Alzheimer Disease Research Centre, Taub Institute), New York, USA, and Lund University (Elisabet Englund, Dnr 286–2014), Lund, Sweden. Experiments were performed in accordance with the guidelines approved by Department of Experimental Medical Science at Lund University, Lund, Sweden and Department of Biology and Biological Engineering at Chalmers University of Technology, Göteborg, Sweden. All samples were taken from the prefrontal cortex, more specifically from *gyrus rectus*. The brain samples were fixed with formaldehyde solution, which has been shown not to interfere with the CARS signal^56^, sectioned into 40 μm thick slices and kept in phosphate buffered saline (PBS). Control samples were labeled with Thioflavin-S or with Cy2 conjugated antibodies against β-amyloid. For CARS microscopy measurements, human brain slices were mounted in a compartment, containing PBS, formed by a spacer between two coverslips secured with double adhesive tape.

### Laser setup

The laser source consists of a Nd:YVO_4_ pump laser (picoTRAIN, HighQ GmbH) and an optical parametric oscillator (OPO), (Levante Emerald OPO, APE GmbH). The laser has two output beams; (i) the fundamental (1064 nm, used as Stokes beam) and (ii) the frequency doubled (532 nm), characterized by a pulse length of 7 ps and a repetition rate of 76 MHz. The 532 nm beam pumps the OPO, providing a variable output wavelength in the range of 690–990 nm (used as pump/probe beam). The OPO output was spatially overlapped with the 1064 nm beam using a dichroic mirror (DMSP1000, Thorlabs). A delay line in the 1064 nm beam path was used for temporal overlap of the excitation beams.

### Microscope setup

Spatially and temporally combined excitation beams were coupled into the mirror scanning unit (Nikon C1) of the confocal microscope (Nikon Eclipse TE2000-E) and focused onto the sample by a high-NA objective (Nikon Plan Fluor 40x/1.30 oil). Forward propagating CARS emission was collected with a high-NA lens, passed the filter block and was focused on a single–photon-counting photomultiplier tube (PMC-100-20, Becker & Hickl GmbH). Epi-propagating fluorescence was collected by the excitation objective and directed by dichroic mirror to the back-port of the microscope. After passing the filter block the fluorescence emission was focused on a single-photon-counting photomultiplier tube (PMC-100-1, Becker & Hickl GmbH).

### CARS and fluorescence microscopy

In the current study the Stokes beam was fixed at 1064 nm, while the pump/probe beam was tuned in the 807–825 nm range. When the energy difference between the Stokes and pump/probe beams matched the energy of a C-H vibrational mode, an enhanced coherent emission from the acyl chain in lipids was generated at the anti-Stokes wavelength of the pump/probe beam. The CH_2_ stretch vibration at 2840 cm^−1^ was probed by tuning the OPO to 817 nm. The forward propagating CARS signal was separated from the excitation light and the fluorescence by four band-pass filters (650/50 nm, Chroma Technologies). Simultaneously, the multi-photon excitation fluorescence (MPEF) from labeled samples was detected in the epi-direction, spectrally selected by optical filters: for Thioflavin S we used a combination of short-pass filters (600SP and 2 × 750SP, Ealing) and for Cy2 we used a band-pass filter (525/50 nm, Chroma). Z-stacks were acquired with a distance of 1 μm, forming 3D images of the AD brain tissues. Single images were obtained with acquisition times of 20 s. Images were formed in ImageJ^57^ by color-coding the number of detected photons in each pixel.

### CARS spectroscopy

For spectral analysis of the CH-stretching vibrational region, the OPO was tuned from 825.2 nm to 807.9 nm, corresponding to the vibrational range from 2720 cm^−1^ to 2980 cm^−1^, in steps of 5 cm^−1^.

### Raman microscopy

For Raman measurements, unstained human AD brain samples were mounted on a cover slip and dried in air. Raman micro-spectroscopy measurements were conducted using a confocal Raman microscope (inVia Raman, Renishaw) with a continuous wave laser source (532 nm). Single Raman spectra were acquired in extended spectral scanning mode (500–3000 cm^−1^) with a 2400 mm^−1^ grating, using a 100x objective, 25 mW excitation power at the sample position and a 10 s integration time.

### Analysis of Raman spectra

Spectral data from Raman microscopy experiments was analyzed using in-house written Matlab routines. All spectra were interpolated to a uniform frequency separation of 1 cm^−1^, baseline corrected using the airPLS algorithm^58^ and smoothed with 7 points moving average filter.

## Additional Information

**How to cite this article**: Kiskis, J. *et al.* Plaque-associated lipids in Alzheimer's diseased brain tissue visualized by nonlinear microscopy. *Sci. Rep.* **5**, 13489; doi: 10.1038/srep13489 (2015).

## Supplementary Material

Supplementary Information
