# Supplementary material for: Plaque-associated lipids in Alzheimer’s diseased brain tissue visualized by nonlinear microscopy
Source: Sci Rep. 2015 Aug 27;5:13489. doi: 10.1038/srep13489 (PMC4550829; doi:10.1038/srep13489)
Supplement: Supplementary Information [file srep13489-s1.pdf]

Plaque-associated lipids in Alzheimer's diseased brain tissue visualized by nonlinear microscopy

Juris Kiskis[1], Helen Fink[1], Lena Nyberg[1], Jacob Thyr[2], Jia-Yi Li[3], and Annika Enejder[1*]

[1] Department of Biology and Biological Engineering, Chalmers University of Technology, Göteborg, Sweden

[2] K-Analys, Uppsala, Sweden

[3] Department of Experimental Medical Science, Lund University, Lund, Sweden

[*] corresponding author

SUPPLEMENTARY INFORMATION

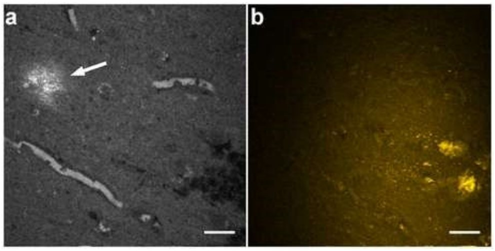

Figure 1S. Visualization of Aβ and lipid distribution in a diffuse plaque by fluorescence labeled with the antibody against 17-24 amino acids of Aβ and CARS microscopy. **(a)** Cy2 fluorescence, arrow indicating a diffuse plaque; (**b**) CARS emission at 2840 cm^{-1}. Scale bars, 25 μm.
